# Collagenase-resistant collagen promotes mouse aging and vascular cell senescence

**DOI:** 10.1111/acel.12155

**Published:** 2013-09-19

**Authors:** Faran Vafaie, Hao Yin, Caroline O’Neil, Zengxuan Nong, Alanna Watson, John-Michael Arpino, Michael W A Chu, David Wayne Holdsworth, Robert Gros, J Geoffrey Pickering

**Affiliations:** 1 Robarts Research Institute, Western UniversityLondon, ON, Canada; 2 Departments of Medicine and Biochemistry, Western UniversityLondon, ON, Canada; 3 Department of Medical Biophysics, Western UniversityLondon, ON, Canada; 4 Department of Surgery, Western UniversityLondon, ON, Canada; 5 Department of Physiology and Pharmacology, Western UniversityLondon, ON, Canada; 6 London Health Sciences CentreLondon, ON, Canada

**Keywords:** collagen, integrin, senescence, vascular smooth muscle

## Abstract

Collagen fibrils become resistant to cleavage over time. We hypothesized that resistance to type I collagen proteolysis not only marks biological aging but also drives it. To test this, we followed mice with a targeted mutation (*Col1a1^r/r^*) that yields collagenase-resistant type I collagen. Compared with wild-type littermates, *Col1a1^r/r^* mice had a shortened lifespan and developed features of premature aging including kyphosis, weight loss, decreased bone mineral density, and hypertension. We also found that vascular smooth muscle cells (SMCs) in the aortic wall of *Col1a1^r/r^* mice were susceptible to stress-induced senescence, displaying senescence-associated ß-galactosidase (SA-ßGal) activity and upregulated p16^INK4A^ in response to angiotensin II infusion. To elucidate the basis of this pro-aging effect, vascular SMCs from twelve patients undergoing coronary artery bypass surgery were cultured on collagen derived from *Col1a1^r/r^* or wild-type mice. This revealed that mutant collagen directly reduced replicative lifespan and increased stress-induced SA-ßGal activity, p16^INK4A^ expression, and p21^CIP1^ expression. The pro-senescence effect of mutant collagen was blocked by vitronectin, a ligand for αvß3 integrin that is presented by denatured but not native collagen. Moreover, inhibition of αvß3 with echistatin or with αvß3-blocking antibody increased senescence of SMCs on wild-type collagen. These findings reveal a novel aging cascade whereby resistance to collagen cleavage accelerates cellular aging. This interplay between extracellular and cellular compartments could hasten mammalian aging and the progression of aging-related diseases.

## Introduction

It has been recognized for over 40 years that collagen becomes progressively stabilized with age (Hamlin & Kohn, 1971). This age-related stabilization of collagen entails increasing intermolecular and interfibril cross-links and a concomitant decline in the susceptibility of collagen to collagenase digestion (Davis *et al*., 1975; Zwolinski *et al*., 1976; Vater *et al*., 1979; Monnier *et al*., 2005). The decline in sensitivity to collagenolytic enzymes appears to be a highly programmed event. For example, digestibility of a collagen sample has been reported to predict the chronological age of an individual (Hamlin & Kohn, 1971, 1972).

Type I collagen is a major component of large- and medium-sized arteries and accumulates in aging blood vessels (Lakatta & Levy, 2003). Vascular aging is central to cardiovascular disease, and growing evidence suggests the process is characterized not only by changes in extracellular matrix proteins but also by aging of the cells resident in the artery wall. Vascular smooth muscle cells (SMCs) are dominant in the artery wall, and their potential for accelerated aging is recognized by their behavior *in vitro*, where a senescence phenotype develops with replication and in response to stress (Kunieda *et al*., 2006; van der Veer *et al*., 2007; Herbert *et al*., 2008; Ho *et al*., 2009). SMCs with features of senescence have also been identified in atherosclerotic arteries (Minamino *et al*., 2003; Kunieda *et al*., 2006; Matthews *et al*., 2006) and in aortas of aged rodents (Yang *et al*., 2007). It is proposed that senescent SMCs contribute to vascular dysfunction through pro-inflammatory signals, perturbed contractility, increased stiffness, and an inability to replicate and repair damaged regions of the artery (Yildiz, 2007; Qiu *et al*., 2010; Wang *et al*., 2010).

Vascular aging thus proceeds in both cellular and extracellular compartments. However, the extent to which cellular and extracellular aging are independent processes vs. functionally linked events is unclear. Of particular interest is the possibility that aging of collagen fibrils might impact aging of SMCs. Such interplay could have substantial consequences for the rate of vascular deterioration. Type I collagen proteolysis has been found to impact SMC adhesion and migration (Carragher *et al*., 1999; Li *et al*., 2000; Fera *et al*., 2004; Nong *et al*., 2011). Whether collagen that is resistant to cleavage affects SMC aging is not known.

To determine whether degradation-resistant collagen impacts aging, we took advantage of mice expressing a collagenase-resistant form of type I collagen (*Col1a1*^*r/r*^). These mice have targeted substitutions in the *Col1a1* gene such that the type I collagen triple helix cannot undergo ¾-¼ cleavage (Wu *et al*., 1990; Liu *et al*., 1995). Because of an additional collagenase cleavage site, these mice are viable, but collagen turnover has been found to be suboptimal during active tissue remodeling (Liu *et al*., 1995). We report that degradation-resistant type I collagen produces a premature aging syndrome and promotes vascular SMC senescence. The findings point to a paradigm whereby the state of the extracellular matrix regulates aging and can drive cellular senescence.

## Results

### *Col1a1*^*r/r*^ mice have shortened lifespan

**Figure 1:**
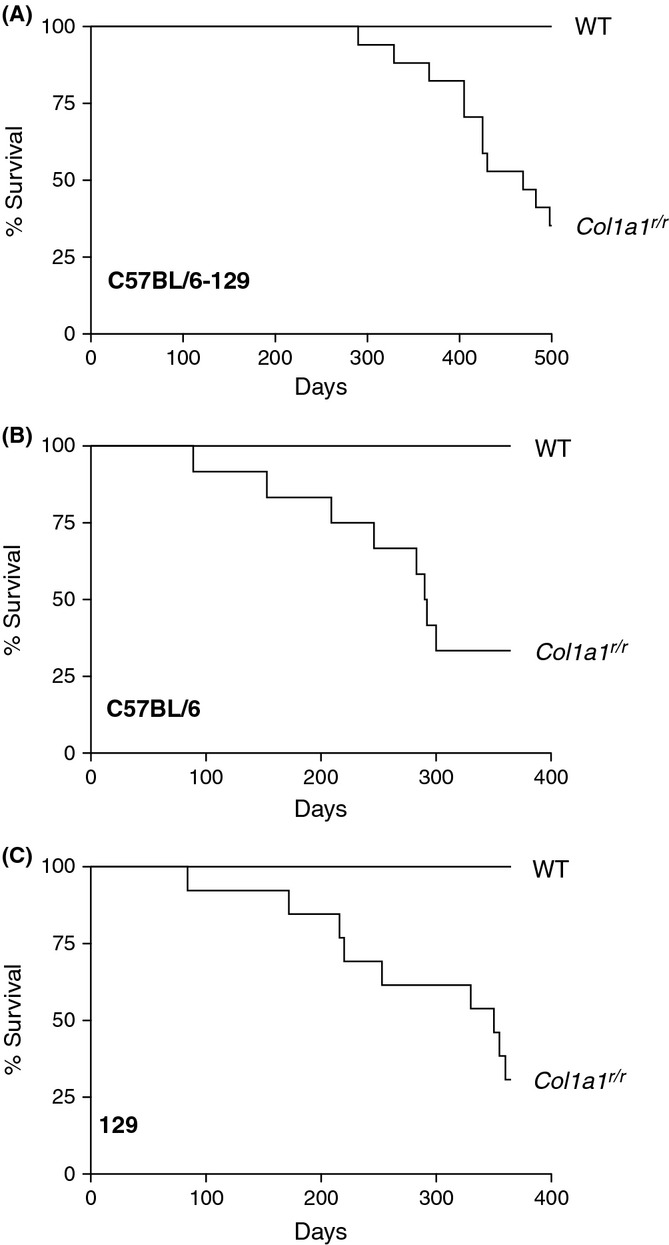
*Col1a1^r/r^* mice have shortened lifespan. Kaplan–Meier survival curves of male wild-type (WT) and *Col1a1^r/r^* mice on three different genetic backgrounds. (A) Survival of mixed C57BL/6-129 mice; *n* = 16 and 17 for WT and *Col1a1^r/r^* *mice*, respectively. *P* < 0.001. (B) Survival of C57BL/6 mice; *n* = 11 and 12 for WT and *Col1a1^r/r^* mice, respectively. *P* = 0.001. (C) Survival of 129 mice; *n* = 13 and 14 for WT and *Col1a1^r/r^* mice, respectively. *P* = 0.005.

To determine whether collagenase-resistant type I collagen could impact mouse lifespan, *Col1a1*^*r/r*^ mice on a mixed C57BL/6-129 background and their wild-type littermates were followed for 70 weeks. All wild-type mice survived during this period (*n* = 16). Mutant mice survived during the first 39 weeks, but by 70 weeks, 65% (11 of 17) had died (Fig. 1A, *P* < 0.001). To ensure that the lifespan shortening of *Col1a1*^*r/r*^ mice was robustly related to the collagen mutation, we bred *Col1a1*^*r/r*^ mice on to a C57BL/6 background (≥ 10 backcrosses) and also on to a 129P1/ReJ (129) background (≥ 9 backcrosses). In both of these additional genetic background scenarios, survival of mutant mice was impaired (Fig. 1B,C). By 1 year, 67% (8 of 12) of C57BL/6 *Col1a1*^*r/r*^ mice and 64% (9 of 14) of 129 *Col1a1*^*r/r*^ mice died, with no mortality in the respective control mice (*P* = 0.001, *P* = 0.005, respectively). Thus, resistance to collagen cleavage imparted a survival disadvantage.

### *Col1a1*^*r/r*^ mice exhibit several age-related phenotypes

**Figure 2:**
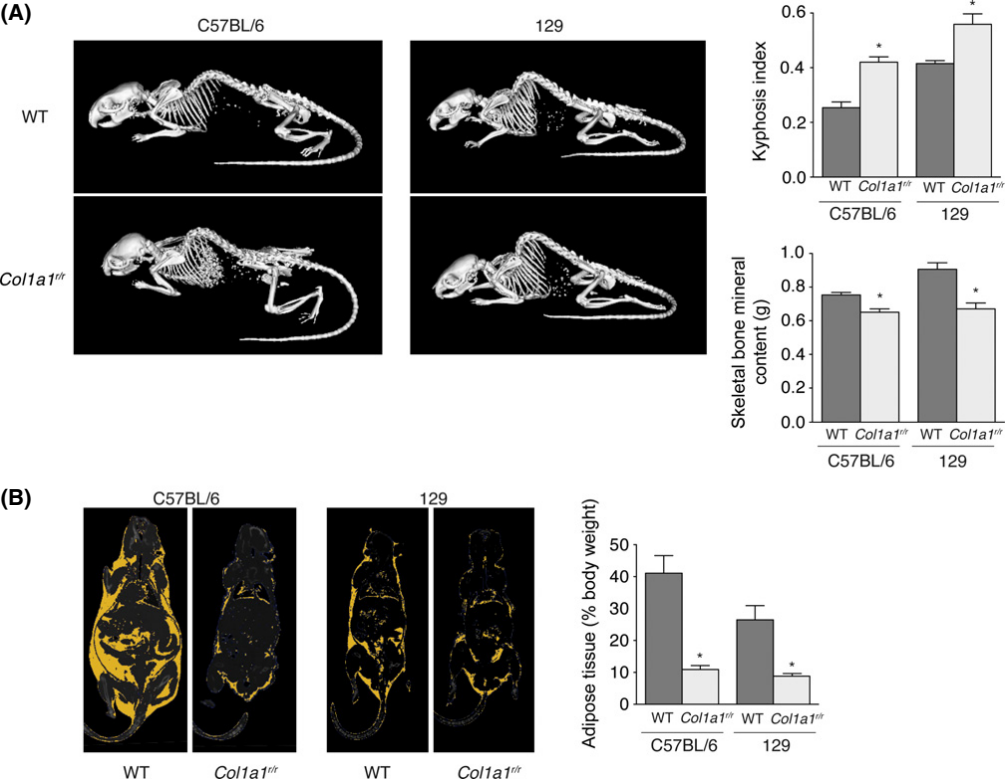
*Col1a1^r/r^* mice exhibit premature aging phenotypes. (A) Three-dimensional surface renderings of micro-CT images of 15.5-month-old C57BL/6 mice and 17-month-old 129 mice. Wild-type and *Col1a1^r/r^* littermates are shown and illustrate increased dorsal kyphosis in the mutant mice. The average kyphotic index of mice at 7–17 months of age (*n* = 7 WT, 8 mutant) is shown in the bar graph on the right. *P = 0.011 for C57BL/6 mice, *P* = 0.007 for 129 mice. Skeletal bone mineral content is reduced in *Col1a1^r/r^* 129 mice, as shown in the graph. *P < 0.001. (B) Micro-CT image depicting adipose tissue (yellow) of wild-type and *Col1a1^r/r^* littermate pairs of C57BL/6 and 129 mice. The bar graph shows the average proportion of adipose tissue (% body weight) of wild-type and *Col1a1^r/r^* mice. *P = 0.006 for mice on the C57BL/6 background, *P* = 0.004 for mice on the 129 background.

No single phenotype at the time of death was observed. Prior to death, some mice showed lethargy, others had skin lesions, but many died suddenly without heralding features. Serum creatinine, urea, and alanine and aspartate aminotransferase levels were not found to be elevated in mice up to 17 months of age, suggesting renal or liver disease was not a prominent feature, if at all (data not shown). However, *Col1a1*^*r/r*^ mice displayed several features of premature aging. Wild-type and mutant mice were the same weight at birth, but by 5 months, *Col1a1*^*r/r*^ mice weighed significantly less than wild-type mice (22.9 ± 0.5 g (*n* = 14) vs. 26.9 ± 0.6 g (*n* = 28) *P* < 0.001). We also noted progressive accentuation of dorsal kyphosis in *Col1a1*^*r/r*^ mice, a characteristic of aging (Kuro-o *et al*., 1997) (Fig. 2). To quantify this, *Col1a1*^*r/r*^ mice and wild-type littermates (average age 12.9 months) on either C57BL/6 or 129 backgrounds underwent micro-CT imaging. Kyphosis measured as the ratio of the maximum height of the spinal curvature to the distance between the 7th cervical and 6th lumbar vertebrae was 39% and 26% greater in C57BL/6 mutant mice and 129 mutant mice, respectively, compared with control mice (*P* = 0.012 and *P* = 0.003, respectively, Fig. 2).

Kyphosis in humans is associated with osteoporosis. To ascertain whether collagenase-resistant collagen leads to osteoporosis, micro-CT imaging was used to quantify bone mineral content using a validated signal intensity threshold strategy (Granton *et al*., 2010). Interestingly, despite the potential for increased bone in the setting of impaired type I collagen catabolism, aged *Col1a1*^*r/r*^ mice on both C57BL/6 and 129 backgrounds had significantly lower bone mineral content than wild-type mice (*P* = 0.034 and 0.002, respectively, Fig. 2).

We also elucidated total fat content by quantitative micro-CT analysis. This revealed that *Col1a1*^*r/r*^ mice had significantly lower adipose content for both C57BL/6 (*P* = 0.006) and 129 strains (*P* = 0.004; Fig. 2). Histologic assessment of skin of aged (11–18 months) mice revealed that not only was the subdermal adipocyte layer significantly thinner in *Col1a1*^*r/r*^ mice (241 vs. 644 μm, *P* < 0.001), the adipocytes themselves were substantially smaller (570 vs. 2466 μm^2^ *P* < 0.001). Because C57BL/6 normally mice accumulate fat with age, the relative fat loss in *Col1a1*^*r/r*^ mice on this background implicates a pathological form of aging imposed by the collagen mutation.

In summary, mutation of the collagenase cleavage site in type I collagen leads to several age-related abnormalities including weight loss, loss of adipose tissue, kyphosis, and osteoporosis. Together with the premature death of *Col1a1*^*r/r*^ mice, these data indicate that reduced type I collagen catabolism imparts a progeroid phenotype in mice.

### *Col1a1*^*r/r*^ mice exhibit age-related hypertension and increased aortic collagen

**Figure 3:**
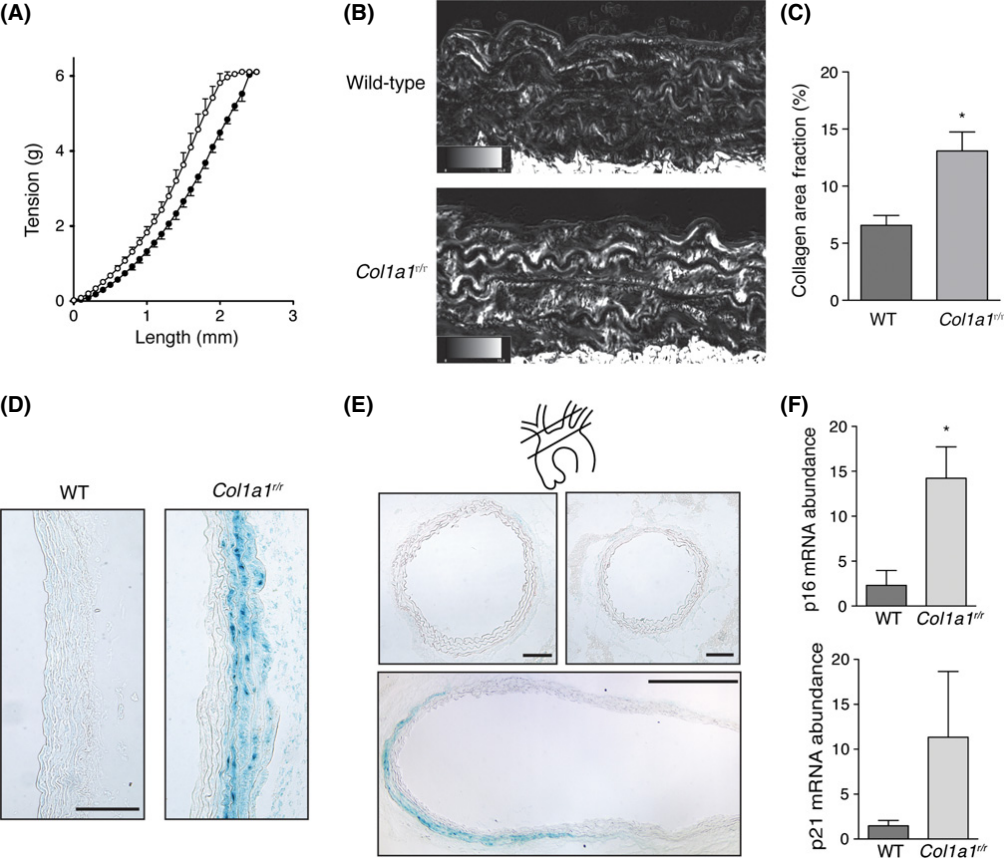
Aortic mechanics, fibrillar collagen content, and susceptibility to smooth muscle cell (SMC) senescence in *Col1a1^r/r^* mice. (A) Length–tension relationships of thoracic aortic ring segments from wild-type (closed circles, *n* = 4) and *Col1a1^r/r^* (open circles, *n* = 5) 13-month-old mice. *P* < 0.001. (B) Microscopic images of sections of the descending thoracic aorta from 15-month-old mice stained with picrosirius red and imaged with circularly polarized light. Birefringent collagen fibrils appear bright. The area fraction of birefringent collagen within the media is depicted in the graph in (C) Data in the graph are from three littermate pairs. *P = 0.010. (D) Light micrographs of the ascending aorta of wild-type and *Col1a1^r/r^* 13-month-old mice, harvested after 28 days of Ang II infusion and stained for senescence-associated ß-galactosidase (SA-ßGal) activity. Intense staining is evident in the outer medial layers of *Col1a1^r/r^* mice, but not in the aorta of wild-type mice. Bar, 100 μm. (E) Micrographs of sections of the brachiocephalic artery (upper left; bar, 100 μm), left carotid artery (upper right; bar, 100 μm), and a composite of the aortic arch (lower; bar, 500 μm). SA-ßGal activity is present in the outer medial layers of the aortic arch, with no activity in the proximal brachiocephalic or left carotid arteries. Section planes are depicted in the schematic. (F) Graphs depicting quantitative PCR assays for mRNA abundance of p16^INK4A^ (upper) and p21^CIP1^ (lower) in thoracic aorta from Ang II-infused mice. Data are from 3 experiments. *P = 0.036.

Another hallmark of aging is hypertension. We previously found that the blood pressure of young (<4 months) *Col1a1*^*r/r*^ mice was not different than that of wild-type control mice (Nong *et al*., 2011). To determine whether differences existed with aged mice, blood pressures of 13-month-old mice were assessed using computerized tail-cuff plethysmography. This revealed significantly higher systolic (141 ± 3 vs. 113 ± 6 mm Hg, *P* < 0.001) and diastolic (99 ± 3 vs. 82 ± 4 mm Hg, *P* = 0.005) blood pressures in *Col1a1*^*r/r*^ mice. Heart rates did not differ among the mice (*P* = 0.567), suggesting no overt differences in sympathetic tone. However, blood pressure assessment revealed a widened pulse pressure in the mutant mice (43 ± 5 vs. 31 ± 9 mm Hg, *P* = 0.005), suggesting increased vascular stiffness. This was supported by *ex vivo* length–tension analyses of thoracic aortic segments from 13-month-old mice, which revealed a leftward shift in the relationship in segments harvested from *Col1a1*^*r/r*^ mice (*P* < 0.001; Fig. 3A). To determine whether these changes were associated with altered collagen content, we undertook circular polarization microscopic imaging of the aortic wall of 15-month-old mice. This revealed a 2.0-fold increase in birefringent collagen within the media in *Col1a1*^*r/r*^ mice (*P* = 0.010; Fig. 3B,C).

### Aortic SMCs in *Col1a1*^*r/r*^ mice are susceptible to angiotensin II-induced senescence

Having identified aging phenotypes in *Col1a1*^*r/r*^ mice, we were interested in determining whether loss of collagen degradability promoted aging at the cellular level. We specifically investigated the vasculature, in view of the importance of vascular aging to several chronic diseases, the abundance of type I collagen in the artery wall, and the finding of hypertension in aged *Col1a1*^*r/r*^ mice. To determine whether cells within the artery wall were susceptible to premature senescence, mice were subjected to a 28-day infusion of vehicle or angiotensin II (Ang II), and the aorta and great vessels were assessed for senescence-associated ß-galactosidase (SA-ßGal) activity *in vivo*. Chronic Ang II delivery induced a 31.6% increase in systolic blood pressure in wild-type mice and 38.7% increase in mutant mice (*P* = 0.513), with no effect detected on heart rate. There was no evidence for aortic aneurysm or rupture on gross examination. SA-ßGal activity was not evident in the aortic wall of either wild-type mice or mutant mice subjected to vehicle infusion. However, SA-ßGal activity was readily detectable in the media of the thoracic aorta of *Col1a1*^*r/r*^ mice (13 months of age) subjected to Ang II infusion, with little to no staining in wild-type mice (Fig. 3D). Interestingly, SA-ßGal signal in *Col1a1*^*r/r*^ mice was localized to the outer medial layers but not endothelial cells. Also, striking was that the senescence signal was confined to the ascending aorta and the convexity of the aortic arch, with no staining in the proximal innominate artery or left carotid artery (Fig. 3E). Evidence for aortic cell senescence in Ang II-infused *Col1a1*^*r/r*^ mice was also evident by upregulation of p16^INK4A^ (*P* = 0.036 Fig. 3F), with a concordant trend for p21^CIP1^ (*P* = 0.235, Fig. 3F).

These data indicate that (i) collagenase-resistant collagen renders vascular SMCs in the aorta susceptible to stress-induced premature senescence; and (ii) that this is a site-specific response, suggesting aging-prone regions of the aorta.

### Proteolysis-resistant type I collagen promotes senescence of human arterial SMCs

**Figure 4:**
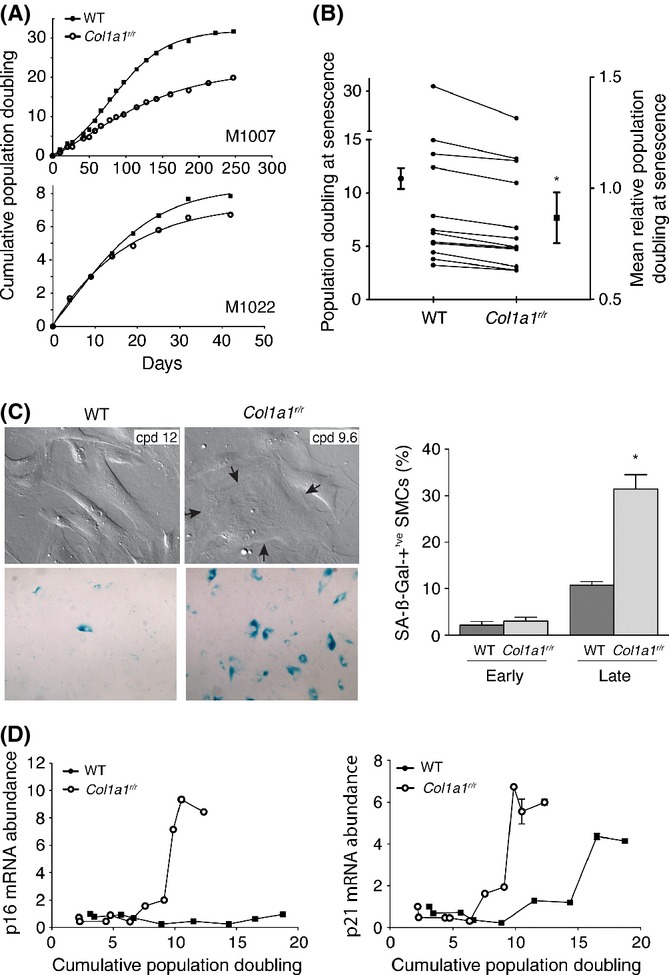
Effect of collagenase-resistant collagen on longevity and replicative senescence of human smooth muscle cells (SMCs). Human vascular SMCs were derived from the internal thoracic artery of patients undergoing coronary bypass surgery. Replicative lifespan and development of senescence was determined for SMCs cultured on mouse tail collagen isolated from wild-type or *Col1a1^r/r^* mice. (A) Cumulative population doubling curves of a long-lived (M1007) and short-lived (M1022) SMC line showing reduced longevity imposed by mutant collagen. (B) Individual and averaged data for SMCs from 12 patients showing replicative lifespan on wild-type and *Col1a1^r/r^* collagen. The mean relative population doubling at senescence was expressed in terms of the lifespan on wild-type collagen for each line. *P* < 0.001. (C) Hoffmann-modulated contrast images (upper panels) showing the emergence of highly spread SMCs on *Col1a1^r/r^* collagen, at relatively early cumulative population doubling compared with those plated on wild-type collagen. A large SMC with thick microfilament bundles and two nuclei is demarcated by the arrows. Light microscope images of parallel cultures stained for SA-ßGal activity (lower panels) show abundant SA-ßGal activity in SMCs on mutant collagen. Quantitative data for the proportion of SA-ßgal-positive SMCs at early (0–5 population doublings) and late (8–13 population doublings) replicative age is shown on the right. *P = 0.003 vs. late-age SMCs on wild-type collagen. (D) Transcript abundance of p16^INK4A^ and p21^CIP1^, quantified by real-time PCR, was serially tracked for SMCs on wild-type or *Col1a1^r/r^* collagen. *P* < 0.001 for both targets.

To explore the mechanism by which proteolysis-resistant collagen promoted SMC senescence, we first asked whether this was a direct or indirect consequence of the degradation-resistant collagen. For this, we cultured human vascular SMCs on collagen harvested from the tails of wild-type and *Col1a1*^*r/r*^ mice. Arterial SMC cultures were derived from 12 patients, and replicative lifespan of each patient-derived line was determined. For SMCs on wild-type collagen, there was a wide range in longevity among the patient lines, ranging from 3.2 to 31.9 population doublings until senescence. However, SMCs from all 12 patients manifested reduced the longevity on mutant collagen, with an average 16.9 ± 10.5% shortening of lifespan (range 5.7–37.4%, *P* < 0.001, Fig. 4A,B). As well, SMCs replicating on *Col1a1*^*r/r*^ collagen were seen to acquire a spread and flattened senescence-like morphology at earlier population doublings than cultures replicating on wild-type collagen (Fig. 4C). Similarly, emergence of SA-ßGal-positive SMCs was accelerated (Fig. 4C). In early subcultures (0–5 population doublings), the proportion of SA-ßGal-positive SMCs was not different for cells on either mutant or wild-type collagen (*P* = 0.480). However, among aged cultures (8–13 population doublings), there was 2.9-fold increase in the proportion of SA-ßGal-positive SMCs on *Col1a1*^*r/r*^ collagen (*P* = 0.003, Fig. 4C).

In addition, serially tracking the transcript abundance of p16^INK4A^ and p21^CIP1^ revealed population doubling-dependent upregulation of both senescence biomarkers was substantially earlier in SMCs on *Col1a1*^*r/r*^ collagen vs. SMCs on wild-type collagen (*P* < 0.001, Fig. 4D).

**Figure 5:**
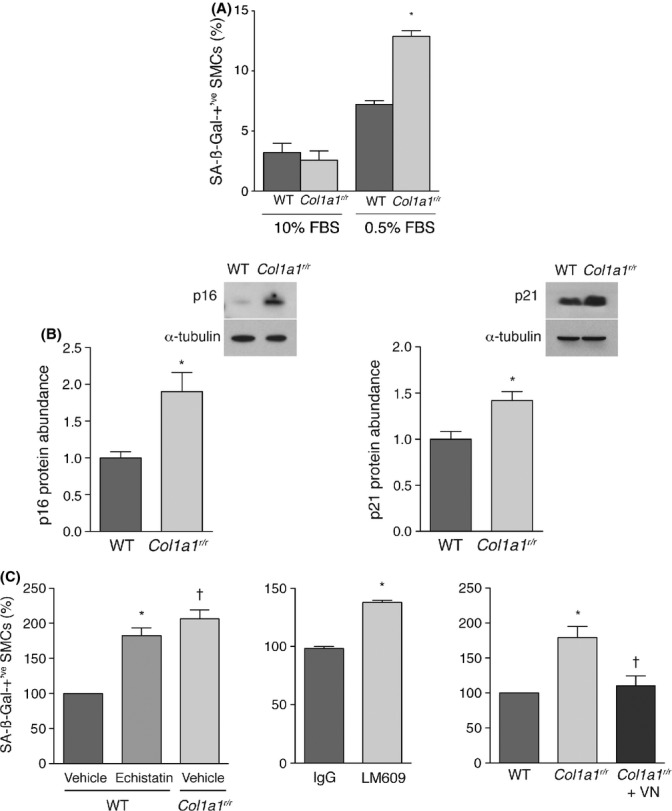
*Col1a1^r/r^* collagen promotes stress-induced premature senescence of smooth muscle cells (SMCs) through insufficient engagement of αvß3 integrin. (A) Graph showing the proportion of SMCs on wild-type or *Col1a1^r/r^* collagen with SA-ßGal activity. Cells were cultured with 10% FBS which was either replenished (*n* = 3) or switched to 0.5% FBS for 3 days to impart to low-serum stress (*n* = 11). *P < 0.001. (B) Representative Western blots and summary graphs showing p16^INK4A^ and p21^CIP1^ mRNA abundance in SMCs on the designated collagen substrate after 3 days of low-serum stress. Data in the graph are from four separate experiments *P = 0.017 for p16^INK4A^, *P = 0.031 for p21^CIP1^. (C) Graphs showing the effects of echistatin (100 nm, left panel), αvβ3 integrin-blocking antibody LM609 (50 μg mL^−1^, middle panel), and vitronectin (50 ng cm^−2^, right panel) on the relative proportion of SMCs in low-serum conditions that displayed SA-ßGal activity. Data are from 3–7 experiments. *P < 0.001 vs. vehicle, ^†^*P* < 0.001 vs. vehicle (left graph); *P = 0.004 (middle graph); *P = 0.015 vs. wild-type collagen, ^†^*P* = 0.003 vs. SMCs on *Col1a1^r/r^* collagen (right graph).

To determine whether *Col1a1*^*r/r*^ collagen impacted stress-induced senescence, SMCs on either wild-type or *Col1a1*^*r/r*^ collagen were subjected to the stress of acute serum withdrawal (10% to 0.5% FBS) for 3 days. For SMCs on wild-type collagen, this induced a 2.3-fold increase (*P* < 0.001) in the proportion of SMCs with SA-ßGal activity and a more striking 5.0-fold increase for SMCs on mutant collagen (*P* < 0.001, Fig. 5A). There were also 1.9- and 1.4-fold increases in abundance of p16^INK4A^ and p21^CIP1^ mRNA for SMCs on mutant collagen under serum-deprivation conditions, respectively, relative to SMCs on WT collagen (*P* = 0.017, *P* = 0.031, respectively, Fig. 5B). In contrast, apoptosis, as assessed by TUNEL assay, was low under these conditions and not different between SMCs on mutant vs. wild-type collagen (0.4 vs 0.6%, *P* = 0.439). Thus, poorly degradable type I collagen imparted senescence signals on both replicating and nonreplicating but stressed SMCs.

### *Col1a1*^*r/r*^ collagen promotes senescence through insufficient engagement of αvß3 integrin

To further assess the mechanism by which mutant collagen promotes cell senescence, we considered the fact that collagen integrin utilization can differ depending on whether uncleaved or cleaved collagen is presented to the cell. Evidence has emerged that whereas α2ß1 integrin is a major receptor for native type I collagen, αvß3 integrin is prominently engaged if type I collagen has been denatured or cleaved (Davis, 1992; Montgomery *et al*., 1994; Li *et al*., 2000; Fera *et al*., 2004). We therefore tested whether blocking αvß3 integrin could mimic the pro-senescence actions of collagenase-resistant collagen, by incubating SMCs on wild-type collagen with the ß3-selective disintegrin, echistatin. This yielded a 2-fold increase in stress-induced SA-ßGal activity compared with vehicle (*P* < 0.001, Fig. 5C), a similar senescence response observed for vehicle-treated SMCs on *Col1a1*^*r/r*^ collagen. The proportion of SMCs with SA-ßGal activity was also increased when SMCs on wild-type collagen were incubated with the αvß3 integrin-blocking antibody, LM609, compared with SMCs incubated with control IgG (*P* = 0.004, Fig. 5D). To determine whether increasing αvß3 integrin signaling could block mutant collagen-induced senescence, SMCs were plated on a substrate of *Col1a1*^*r/r*^ collagen and the αvß3 integrin ligand, vitronectin (50 ng cm^−2^). This abrogated the pro-senescence effect of *Col1a1*^*r/r*^ collagen (*P* = 0.003, Fig. 5E). Together, these data indicate that the senescence stimulus imparted by degradation-resistant type I collagen can be at least partly attributed to insufficient engagement of αvß3 integrin.

### *Col1a1*^*r/r*^ MEFs exhibit enhanced senescence

**Figure 6:**
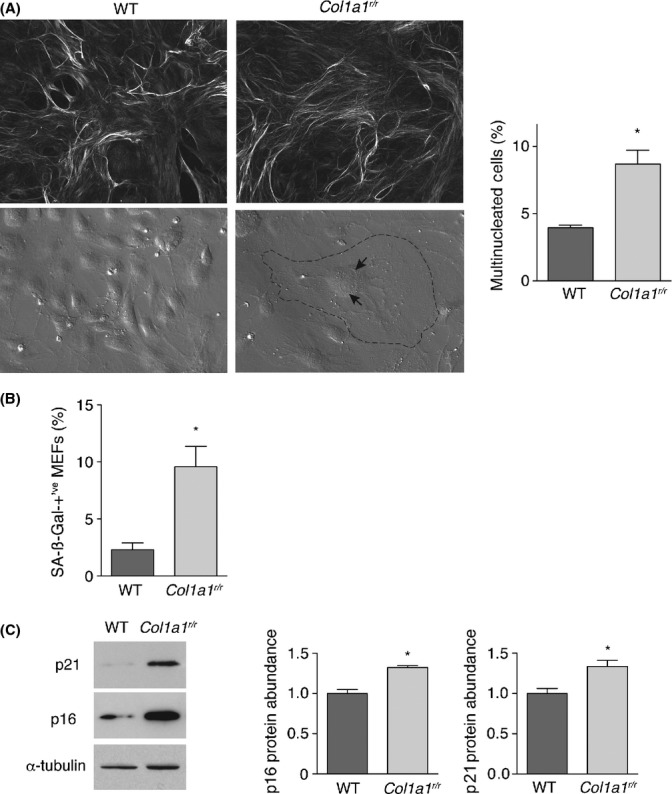
Mouse embryonic fibroblasts (MEFs) from *Col1a1^r/r^* mice exhibit enhanced senescence. (A) *Top* Circular polarization microscopy images of collagen fibril networks elaborated over 14 days by wild-type and *Col1a1^r/r^* MEFs, showing elaborate networks in both cases. Cultures were stained with picosirius red. *Bottom* Hoffman-modulated contrast images of 6th passage wild-type and *Col1a1^r/r^* MEFs, illustrating emergence of large (dashed perimeter line), multinucleated (arrows) *Col1a1^r/r^* MEFs. The proportions of multinucleated MEFs from 3 separate MEF lines from both mouse strains are shown on the right. *P = 0.011. (B) Graph showing the proportion of wild-type and *Col1a1^r/r^* MEFs that display SA-ßGal activity. *P = 0.018. (C) Western blots showing the relative abundance of p16^INK4A^ and p21^CIP1^ in wild-type and *Col1a1^r/r^* MEFs at passage 6, with quantitative data from 3 lines from each mouse strain harvested at passage 5 or 6 shown in the graphs. *P = 0.005 for p16^INK4A^; *P* = 0.014 for p21^CIP1^.

Finally, we evaluated the impact of poorly degradable collagen as an innately elaborated 3-dimensional network, recognizing that signaling from 3-dimensional environments differs from that from 2-dimensional extracellular matrix substrates (Cukierman *et al*., 2001). We therefore isolated mouse embryonic fibroblasts (MEFs) from wild-type and *Col1a1*^*r/r*^ mice and evaluated their program of replicative senescence. To confirm the assembly of a collagen fibril network *in cellulo*, 5th passage subcultures were imaged after 14 days using circular polarization microscopy, which revealed an extensive network of birefringent collagen fibrils elaborated by MEFs derived from both wild-type vs. *Col1a1*^*r/r*^ mice (Fig. 6A). By the 6th subculture, the proportion of MEFs that were multinucleated was 2.2-fold greater in the mutant MEF cultures (*P* = 0.011, Fig. 6A) (Matsumura, 1980). As well, there was a 4.1-fold increase of SA-ßGal-positive cells in mutant MEFs (*P* = 0.018, Fig. 6B), a 32% increase in abundance of 16^INK4A^ (*P* = 0.005) and a 33% increase in p21^CIP1^ (*P* = 0.014, Fig. 6C). Thus, the construction and maintenance of a 3-dimensional collagen fibril matrix requires proteolytic processing of type I collagen to optimize the lifespan of the collagen-producing cell.

## Discussion

The data herein establish that mice expressing cleavage-resistant type I collagen undergo accelerated and pathological aging, with weight loss, reduced adiposity, kyphosis, osteoporosis, hypertension, and premature death. We also established that vascular aging in *Col1a1*^*r/r*^ mice was not confined to the extracellular domain but also entailed cellular aging, with senescence of vascular SMCs. These findings are important because they indicate that poorly degradable type I collagen is not only a consequence of aging but a cause. Moreover, they indicate that cellular aging can be regulated by the state of the extracellular matrix.

*Col1a1*^*r/r*^ mice afforded a unique opportunity to isolate the impact of collagen degradability on aging. The mutated α1(I) collagen chain renders type I collagen resistant to the seminal ¾-¼ cleavage of the type I collagen triple helix (Wu *et al*., 1990; Liu *et al*., 1995). The resulting decrease in collagenase sensitivity thus models the fall in degradability of naturally aged collagen fibrils (Vater *et al*., 1979). Use of *Col1a1*^*r/r*^ mice offered advantages over targeting a collagenase, as there are several interstitial collagenases that can mediate triple helix cleavage (Spinale, 2007). In addition, because collagenases can have targets other than type I collagen (Spinale, 2007), assessing mice in which the collagen was modified ensured that the findings were not confounded by collagenase-mediated cleavage of other proteins. It was also noteworthy that we found progeroid features in *Col1a1*^*r/r*^ mice on three different genetic backgrounds. Collectively these findings strongly support degradation-resistant type I collagen as a driver of accelerated aging.

The aorta is a type I collagen-rich tissue that is subjected to considerable hemodynamic and oxidative stresses. Over time, resulting changes in structure and function can lead to hypertension, atherosclerosis, and aortic rupture. The finding of senescent SMCs in Ang II-infused aortas of *Col1a1*^*r/r*^ mice is noteworthy therefore and to our knowledge the first evidence for widespread senescence of medial SMCs, in the absence of atherosclerosis.

Interestingly, infusion of Ang II induced SMC senescence with little evidence for senescence in endothelial cells. This selectivity might reflect quantitatively greater exposure of SMCs to type I collagen. Also noteworthy was the localization of SMC senescence to the ascending aorta and arch, with relative sparing of SMCs in the distal aorta. The reasons for this spatial heterogeneity are unknown; however, it is recognized that the proximal aorta is susceptible to age-related and atherosclerosis-independent degeneration in humans (Castellano *et al*., 2012). Daugherty and co-workers have recently identified that the response of the proximal aorta to Ang II differs from that of the distal aorta, with SMC hyperplasia observed only proximally (Owens *et al*., 2010). Proliferating SMCs can be expected to increase their contact with interstitial type I collagen-containing fibers as they egress from their basement membrane; this might account for the selective predisposition of proximal aortic SMC senescence in *Col1a1*^*r/r*^ mice. It is also possible that the reactive oxygen species burden imposed by Ang II is amplified in replicating SMCs, heightening the aging stress in the proximal aorta (Touyz & Schiffrin, 2001). Regardless of the specific mechanisms underlying the localization of SMC senescence, the findings strongly suggest that the rate at which activated SMCs in the proximal aorta age depends on the extent to which type I collagen can be proteolytically modified.

It is likely that several organs are impacted over time by the presence of poorly degradable collagen, and SMC senescence is unlikely to be the sole basis for accelerated aging and premature death in *Col1a1*^*r/r*^ mice. Similarly, we cannot exclude the possibility that SMCs in the aorta of *Col1a1*^*r/r*^ mice were senescent as a result of systemic perturbations, for example metabolic or hemodynamic changes from dysfunction of organs other than the aorta. However, we did not identify serum biomarkers of renal or liver failure. Furthermore, a direct pro-aging effect of nondegradable collagen was supported by several *in vitro* findings. First, human SMCs plated on collagenase-resistant collagen displayed shortened replicative lifespan. This was a reproducible response that was observed in SMCs from 12 different patients. Second, *Col1a1*^*r/r*^ collagen also induced replication-independent, stress-induced senescence of cultured SMCs. Finally, MEFs derived from *Col1a1*^*r/r*^ mice underwent accelerated aging as they endogenously produced and assembled a 3-dimensional collagen fibril-rich matrix, a physiological means of presenting collagen to the cell.

We propose that one basis by which type I collagen turnover determines cell longevity lies in its capacity to redirect cell signaling. Native type I collagen interacts with SMCs through ß1 integrins, including α2ß1 integrin (Pickering *et al*., 1997). However, upon collagenase-mediated cleavage, the triple helix unwinds to expose cryptic RGD sites that serve as ligands for ß3 integrins including αvß3 integrin, which has anti-apoptotic properties (Davis, 1992; Montgomery *et al*., 1994). Interestingly, we found that inhibiting αvß3 integrin signaling in SMCs mimicked the pro-senescence effect of collagenase-resistant type I collagen, with no observed effect on apoptosis. Consistent with this, induction of cell senescence by *Col1a1*^*r/r*^ collagen was abrogated by the αvß3 ligand, vitronectin. Importantly, although widespread collagen degradation can disrupt vessel integrity, SMCs have the capacity to cleave type I collagen in a highly localized and orchestrated manner, through plasma membrane-bound collagenase-1 (Li *et al*., 2000; Fera *et al*., 2004). The fact that *Col1a1*^*r/r*^ MEFs underwent accelerated senescence without obvious changes to the structure of the collagen network is consistent with localized or subtle collagen modification. We propose that localized editing of type I collagen in the immediate pericellular space enables SMCs, by enhancing αvß3 integrin signaling, to resist aging signals that arise within the vasculature.

It is noteworthy that protease-generated fragments of type I collagen have been shown to cause arterial vasodilation and that this response is also mediated by αvß3 integrin (Mogford *et al*., 1996). This finding supports the functional importance of proteolytically modified type I collagen in the vasculature but also raises the intriguing possibility that reduced αvß3 integrin signaling in SMCs, in addition to vascular stiffening, contributed to the hypertension observed in *Col1a1*^*r/r*^ mice. Although speculative, an age-dependent reduction in type I collagen proteolysis could be a factor that unifies aging and hypertension.

In summary, proteolysis-resistant type I collagen is not only a biomarker of aging but can itself promote aging. We have identified that one mechanism by which collagenase-resistant collagen contributes to accelerated aging is by predisposing vascular SMCs to undergo senescence. In so doing, degradation-resistant collagen effectively extends the burden of tissue aging from the extracellular space to the cellular compartment. This self-amplifying paradigm could hasten biological aging and progression of aging-related diseases. Strategies to minimize age-related changes in type I collagen thus warrant consideration.

## Experimental procedures

### Animals

*Col1a1*^*r/r*^ mice (Liu *et al*., 1995) were generated from heterozygous mice containing a targeted mutation of the *Col1a1* gene (B6,129S4-Col1a1^tm1Jae^/J; The Jackson Laboratory, Bar Harbor, ME, USA). In addition to the mixed-background strain, *Col1a1*^*r/r*^ mice on a C57BL/6J background and 129^P1/ReJ^ background were generated by backcrossing for at least 9 generations. Mice were genotyped by PCR analysis of tail DNA as described (Zhao *et al*., 1999). Serology was quantified using Charles River Biomarkers Service (Wilmington, MA, USA). Blood pressure was measured with tail-cuff plethysmography (CODA, Kent Scientific Corp, Torrington, CT, USA). Measurements were recorded after a 5-day measurement acclimatization schedule.

For infusion of Ang II, mice were anesthetized with ketamine (80 mg kg^−1^) and xylazine (10 mg kg^−1^) and osmotic pumps (Alzet model 2004) delivering either Ang II (Sigma Chemical Co.) or saline at an infusion rate of 0.25 μL h^−1^ for 28 days (1000 ng Ang II/kg min^−1^) were implanted subcutaneously on the flank. Animal experiments were undertaken using male mice and performed in accordance with the Canadian Guide for the Care and Use of Laboratory Animals.

### Micro-CT imaging

Animals were scanned using a cone-beam, volumetric, micro-CT scanner (GE eXplore Locus Ultra, GE Healthcare, Waukesha, WI, USA). CT imaging is capable of differentiating body composition based on gray-scale intensity (Hounsfield Units) of measured voxels, due to differences in tissue density. Three gray-scale thresholds were generated corresponding to lean tissue, adipose tissue and bone, to assign each voxel to a tissue type. Tissue volume was determined by voxel number (voxel volume = 0.0037 mm^3^). Bone mineral content was calculated based on the known linear relationship between CT number and mineral content, and using calibration samples containing known amounts of bone mineral equivalent densities, as described (Granton *et al*., 2010).

Kyphotic index of mice was obtained from the micro-CT skeletal structure images by measuring the linear distance from the 7th cervical to the 6th lumbar vertebrae (D1) and the vertical distance from a point along that line to the vertebral body corresponding to the apex of the spinal curvature (D2). The kyphotic index was expressed as D2/D1, wherein that the higher the ratio the greater the kyphosis.

### Aortic stiffness assessment

Aortic stiffness was assessed by generating *ex vivo* length–tension curves. Aortic segments were excised and placed in ice-cold Krebs solution. Two-millimeter aortic ring segments with adventitia removed were mounted isometrically on two parallel stainless steel wires connected to a force transducer (FT03; Grass Instruments, Warwick, RI, USA). Following preconditioning, the aortic rings were equilibrated at zero tension, and length–tension curves were generated by increasing the distance from the zero tension position by 100 μm, every 2 min.

### Cell culture

Primary cultures of human vascular SMCs were derived from internal thoracic arteries of patients undergoing coronary artery bypass surgery, as previously described (Pickering *et al*., 1992; Isner *et al*., 1994). MEFs were isolated as previously described (Xu, 2005) from wild-type and *Col1a1*^*r/r*^ E13.5 embryos. To determine replicative lifespan, cells plated at 4500 cells cm^−2^ were grown to 90% confluence and serially subcultured until cessation of growth. Cumulative population doublings (CPD) were assessed as reported previously (Frontini *et al*., 2011). Apoptosis was assessed by fluorescence *in situ* end-labeling of DNA fragments (TUNEL Label, Roche).

### Senescence-associated β-galactosidase activity assay

SA-ßGal activity in the mouse aorta and great vessels was determined by antegrade perfusion via the left ventricle with phosphate-buffered saline (PBS) followed by perfusion with SA-ßGal staining solution (1 mg mL^−1^ X-Gal, 40 mm sodium citrate, 5 mm potassium ferrocyanide, 5 mm potassium ferricyanide, 150 mm NaCl, 2 mm MgCl_2_, adjusted to pH 6.0 with 1 m sodium phosphate, monobasic). Aortas were then harvested, incubated in SA-ßGal staining solution at 37 °C for 8 h, fixed in 4% paraformaldehyde for 6 h, and cryosectioned at 10 μm. SA-ßGal activity was identified based on positively stained blue cells.

To assess SA-ßGal activity in cultured SMCs, cells were fixed in 2% formaldehyde and 0.2% glutaraldehyde in PBS for 3 min and incubated in X-gal staining solution at 37 °C for 16 h. To assess SA-ßGal activity in MEFs, cells were fixed with 0.5% glutaraldehyde in PBS for 10 min and incubated overnight at 37 °C in modified SA-ßGal staining solution (0.5 mg mL^−1^ X-gal, 0.12 mm potassium ferrocyanide, 0.12 mm potassium ferricyanide, 1 mm MgCl_2_ in PBS, adjusted to pH 6.0) (Yang *et al*., 2000).

### Purification of mouse type I collagen and extracellular matrix coating

Type I collagen was harvested from the tails of *Col1a1*^*r/r*^ and wild-type mice, as described (Rocnik *et al*., 2001). Collagen yield from mutant mice was 50–70% lower than that from wild-type mice. To coat culture substrates with collagen, glass coverslips were pretreated with 0.1% acetic acid and then incubated for 3 h at room temperature with mouse tail collagen at 0.1 mg mL^−1^ in 0.1% acetic acid, washed three times with PBS, and once with culture medium. Plating efficiency, determined by picrosirius red staining of coverslips, a standard curve derived from known amounts of collagen air-dried onto coverslips, and quantification using Imagej, was not different for wild-type or mutant collagen (22 ± 3 vs. 20 ± 3% *P* = 0.290, *n* = 4). To coat dishes with vitronectin and tail collagen, collagen-coated coverslips were incubated with 50 ng cm^−2^ of purified human vitronectin (Millipore, Billerica, MA, USA) at 37 °C for 2 h and washed as above.

### Quantitative real-time reverse transcription–polymerase chain reaction

Aortic tissue and cultured cells were lysed with Trizol (Invitrogen, Burlington, ON, Canada), and total RNA was extracted using the Qiagen RNeasy Kit (Qiagen, Mississauga, ON, Canada). RNA concentration was quantified using NanoDrop ND-1000 (Thermo Scientific, Mississauga, ON, Canada). cDNA was synthesized with Multiscribe Reverse Transcriptase (Applied Biosystems, Streetsville, ON, Canada). Transcript abundance of human and mouse p16^INK4A^, p21^CIP1^, and GAPDH were assessed with Taqman-based primer/probe sets (Applied Biosystems) and ABI 7900HT Fast Real-time PCR apparatus and Sequence Detection System software. Quantification of relative mRNA abundance based on critical threshold (CT) was assessed using the comparative CT formula, 2-ΔΔCT, with GAPDH mRNA as an internal control.

### Western blot analysis

Western blot analysis was undertaken with chemiluminescent detection as previously described (Frontini *et al*., 2009). Blots were probed by incubating with primary antibodies reacting to the following: human and mouse p21^CIP1^ (sc-756, Santa Cruz, 1:1000); human p16^INK4A^ (51-1325GR, 1:500; BD Biosciences, Mississauga, ON, Canada); mouse p16^INK4A^ (sc-1207, Santa Cruz, 1:500); and human and mouse α-tubulin, 1:30 000 (T5168; Sigma-Aldrich, Oakville, ON, Canada).

### Visualization of collagen fibrils elaborated *in cellulo*

Collagen fibrils in paraffin-embedded sections of the aortic media, and elaborated by MEFs in culture, were visualized using circular polarization microscopy (Whittaker *et al*., 1994; Pickering *et al*., 1996; Nong *et al*., 2011). MEFs were fixed with 4% paraformaldehyde for 20 min and collagen fibrils stained with picrosirius red (Polysciences, Warrington, PA, USA). Images were acquired using an Olympus BX51 microscope equipped with Olympus BX series circular polarizer/interference filters, a liquid crystal compensator and CCD camera, and processing software (Abrio LC-PolScope, Cambridge Research & Instrumentation, Woburn, MA, USA).

### Statistical analysis

Values are expressed as mean ± standard error of the mean. Statistical analyses were performed using GraphPad Prism software (GraphPad, La Jolla, CA, USA). Student’s *t*-test or one-way anova was used for comparing mean data from separate experiments. Kaplan–Meier survival analysis was used to assess mouse longevity, and data were compared using log-rank and Wilcoxon testing. Cumulative replicative lifespan plots of cultured SMCs were compared using Wilcoxon matched pairs testing. Transcript abundance of p16^INK4A^ and p21^CIP1^ throughout replicative aging, and the aortic length–tension data, were compared using two-way anova.
